# The “Universal” in UHC and Ghana’s National Health Insurance Scheme: policy and implementation challenges and dilemmas of a lower middle income country

**DOI:** 10.1186/s12913-016-1758-y

**Published:** 2016-09-21

**Authors:** Irene Akua Agyepong, Daniel Nana Yaw Abankwah, Angela Abroso, ChangBae Chun, Joseph Nii Otoe Dodoo, Shinye Lee, Sylvester A. Mensah, Mariam Musah, Adwoa Twum, Juwhan Oh, Jinha Park, DoogHoon Yang, Kijong Yoon, Nathaniel Otoo, Francis Asenso-Boadi

**Affiliations:** 1 Ghana Health Service, Research and Development Division, P.O. Box 1, Dodowa, Greater Accra Region Ghana; 2 Department of Social and Behavioral Sciences, University of Ghana, School of Public Health, Accra, Ghana; 3 Department of Epidemiology, University of Ghana, School of Public Health, Accra, Ghana; 4 Policy Analysis Unit, Policy Planning Monitoring and Evaluation Division, Ministry of Health, Accra, Ghana; 5 Korea Foundation for International Healthcare (KOFIH), Seoul, Republic of Korea; 6 National Health Insurance Authority, Accra, Ghana; 7 JW LEE Center for Global Medicine, Seoul National University College of Medicine, Seoul, Republic of Korea; 8 National Health Insurance Service, Seoul, Republic of Korea

**Keywords:** Universal Health Coverage, Policy, Implementation, National Health Insurance Scheme, Ghana, Low and middle income countries

## Abstract

**Background:**

Despite universal population coverage and equity being a stated policy goal of its NHIS, over a decade since passage of the first law in 2003, Ghana continues to struggle with how to attain it. The predominantly (about 70 %) tax funded NHIS currently has active enrolment hovering around 40 % of the population. This study explored in-depth enablers and barriers to enrolment in the NHIS to provide lessons and insights for Ghana and other low and middle income countries (LMIC) into attaining the goal of universality in Universal Health Coverage (UHC).

**Methods:**

We conducted a cross sectional mixed methods study of an urban and a rural district in one region of Southern Ghana. Data came from document review, analysis of routine data on enrolment, key informant in-depth interviews with local government, regional and district insurance scheme and provider staff and community member in-depth interviews and focus group discussions.

**Results:**

Population coverage in the NHIS in the study districts was not growing towards near universal because of failure of many of those who had ever enrolled to regularly renew annually as required by the NHIS policy. Factors facilitating and enabling enrolment were driven by the design details of the scheme that emanate from national level policy and program formulation, frontline purchaser and provider staff implementation arrangements and contextual factors. The factors inter-related and worked together to affect client experience of the scheme, which were not always the same as the declared policy intent. This then also affected the decision to enrol and stay enrolled.

**Conclusions:**

UHC policy and program design needs to be such that enrolment is effectively compulsory in practice. It also requires careful attention and responsiveness to actual and potential subscriber, purchaser and provider (stakeholder) incentives and related behaviour generated at implementation levels.

**Electronic supplementary material:**

The online version of this article (doi:10.1186/s12913-016-1758-y) contains supplementary material, which is available to authorized users.

## Background

The United Nations General Assembly adopted the Sustainable Development Goals (SDG) at its seventieth session in September 2015. The SDG vision statement for health includes: *“To promote physical and mental health and well being, and to extend life expectancy for all, we must achieve universal health coverage and access to quality health care”* [1]. Universal Health Coverage (UHC) is a means to an end rather than an end in itself. Very simply it strives to “*ensure that all people have access to needed, quality health services without suffering financial hardship*” [2].

Ghana’s current UHC journey began in 2003 when the National Health Insurance Act 650 was passed by parliament. At that time, the policy objectives of the Ghana National Health Insurance Scheme (NHIS) were stated as: “(…) *to assure equitable and universal access for all residents of Ghana to an acceptable quality package of essential healthcare”* [3].

The design of the Ghana NHIS is a single payer model, with a National Health Insurance Fund (NHIF) made of contributions from a 2.5 % Value Added Tax, known as the National Health insurance levy; 2.5 percentage point of the Social Security and National Insurance Trust (SSNIT) contributions of SSNIT contributors; and an out of pocket premium for non SSNIT contributors (mainly but not exclusively informal sector workers) of approximately Ghanaian cedi (GHS) 7.20 – 48.00 (USD 1 = GHS 3.75 as at 4/5/16) per head per annum. About 60 % of Ghana’s population comprising SSNIT contributors and pensioners, those deemed too poor to pay including Livelihood Empowerment Against Poverty (LEAP) beneficiaries (indigents), children less than 18 years of age, persons aged above 70 and pregnant women are exempt from paying out of pocket premiums. However these exempt groups, like the groups that pay out of pocket premiums need to go annually to a district insurance scheme office or community agent to enroll or renew enrollment. Additionally, apart from indigents, LEAP beneficiaries and pregnant women, all subscribers pay an annual registration fee equivalent to approximately USD 2 per head per annum for enrolment and card processing. The LEAP program is a government of Ghana social cash transfer program that provides cash and health insurance to households across the country classified as extremely poor using the program targeting mechanisms [4]. Ninety percent (90 %) of LEAP households have at least one member enrolled in the NHIS [5].

The NHIS was introduced in 2003 as a politically decentralized or devolved district mutual health insurance scheme model, with local government (district assemblies) responsible to set up and oversee the district schemes. Because of concerns about the quality of management and fiscal control, the system was reformed in 2012 with the passage of a revised national health insurance law (Act 852), which created an administratively decentralized or deconcentrated model. The semi-autonomous district mutual health insurance schemes were converted into branch offices of the National Health Insurance Authority (NHIA). The NHIA established regional offices in each of the ten regions of Ghana to oversee the district offices. The new law consolidated a pre-existing tendency to increase central control and move from a devolved to a deconcentrated system of management. The reform enabled tighter central fiscal control of frontline purchaser staff and services. However, to some extent it also distanced services from the District Assemblies (local government). It is not clear this has had any effect on enrolment.

Enrolment in the NHIS has stagnated at about 40 % of the population, after rising rapidly in the early years of its establishment [6]. Even out of pocket premium exempt groups do not all enroll and renew enrolment in the scheme. There are also inequities in enrolment. The Multiple Indicator Cluster Survey 2012 report [7] showed that the percentage of men and women ever registered in the NHIS rises with income from the lowest quintile through to the highest. The 2008 [8], 2014 [9] Ghana Demographic and Health surveys and other work e.g., the SHINE project household survey data on NHIS enrolment in the Central and Eastern regions carried out in 2009 show similar patterns [10]. Routine health management information system and survey data show higher utilization of health care services among the insured as compared to the uninsured. The differences in enrolment between income quintiles translate into differences in utilization that further magnify the inequities under the NHIS. Thus the inequities in voluntary NHIS enrolment translate into inequities in service utilization and access [11]. Beyond income quintiles, survey as well as routine management information system data of the NHIS shows differences in population coverage between regions, demographic groups as well as ‘premium exempt’ versus ‘non-premium exempt’ groups. Given the persisting inequities in enrolment, despite multiple out of pocket premium exempt categories; it would appear getting closer to universal population coverage, rather than targeted exemptions may be a better option for improving the equity and catastrophic protective effect of Ghana’s NHIS.

This study aimed to understand why enrolment within the NHIS has stagnated at the current levels and the implications for NHIS policy and program design and implementation towards universal population coverage. Specifically, we explored who is enrolling and renewing enrolment in the NHIS and reasons, barriers and enablers that explain the observations.

### Review of the literature

Ghana’s National Health Insurance scheme grew out of preceding community based health insurance (CBHI) schemes. Despite innovations such as tax funding through a value added tax, a single pooled national fund, and national health insurance authority with regional and district offices as the purchaser, several design feature such as voluntary enrolment derive from the CBHI model. There are several studies of enrolment in voluntary CBHI schemes in LMIC. Almost all of them show low enrolment and difficulties in attaining universal population coverage. In rural West Africa and Uganda, factors such as institutional rigidities and socio-cultural practices, were documented as playing an important role in shaping the decision to voluntarily enroll [12, 13]. The findings of two recent reviews [14, 15] of enrolment in voluntary insurance schemes in LMIC showed similar clusters of factors affecting enrolment. Factors related to service provision such as poor health care quality including stock outs of medicines and other critical service delivery inputs, long waiting times and poor health worker attitudes were one cluster of problems that affected enrolment. A second cluster was insurance scheme factors such as trust in the scheme, timing and modulation of premium collection and scheme rules. A third cluster was related to clients socio-demographic, economic and other characteristics such as education, gender, income and community knowledge and understanding of social health insurance principles in general and the scheme arrangements in particular.

In Ghana, a few studies have shown similar factors at play in the stagnation of enrolment in the NHIS. Lower socio-economic status and large household sizes have been found to contribute to the inability to afford full insurance for households [16]. In a 2008 citizens’ assessment of the NHIS, 77 % of household respondents, who had not enrolled with the NHIS, cited affordability as the reason [17]. The price of NHIS, provider attitudes, peer pressure and convenience were found to be strongly associated with the decision to enroll voluntarily and retain membership within the Ghana NHIS [10].

To guide our data collection, we drew on this literature, and theorized that factors affecting NHIS enrolment could be classified as related to the purchaser, the provider, the client and context.

## Methods

### Background of the study area

The Volta region, one of the ten regions of Ghana, was purposively selected for the study because the co-sponsor for the study, the Korea Foundation for International Healthcare (KOFIH) was involved in supporting Maternal and Child Health programs in that region. Additionally, it was one of three regions where phase two of the scale up of the NHIS per capita provider payment system for primary care was planned, and findings from the study were considered of relevance to the scale up.

In the 2010 national population census the Volta region had a population of a little over two million, about 8.6 % of Ghana’s population [18]. Its inter censal growth rate of 2.5 % was the same as the national average. Ghana has a relatively young population with 43 % of the population aged less than 18 years and the Volta region is no different.

A municipality and a rural district adjoining each other, were purposively selected to study contrasting contexts of an urbanized district with relatively good health service access and a rural district with poor health service access. For reasons of research ethics and confidentiality, we do not use the names of the districts or the communities in which the study was conducted in this publication.

The Municipality had a population of almost three hundred thousand in the 2010 population and housing census. It had relatively good social, economic and healthcare infrastructure, water and sanitary conditions, and fairly high literacy rates compared to the rest of the region. It had 45 health facilities including a regional and municipal hospital, with 43 run by the Ghana Health Service, one mission and one privately owned. Formal sector economic activity comprised mainly of employment in the public service, private services sector and private construction companies. Non formal sector economic activities were petty trading, subsistence farming, animal rearing, artisans and vocations such as hairdressing and dressmaking. It had an NHIS district scheme office.

The population of the rural district was about 64,404 in the 2010 population and housing census. The district lacked basic social and economic infrastructure such as road networks, telecommunication, industry, banking etc. and had serious problems with access to healthcare, water and sanitation facilities. The main occupations were farming, Kente weaving and petty trading [19]. The Administrative Capital had one health centre with a trained midwife, nurses, dispenser and other supporting staffs. There was no hospital. Other sources of modern biomedical health care in the district were two health centres and several small clinics and Community Health Planning and Services compounds.

### Study design and data collection methods

We employed an exploratory, mixed methods cross sectional case study design. Data collection involved extraction of routine management information system data from the district insurance scheme records, community focus group discussions (FGD) and key informant (KI) interviews. We also reviewed NHIS policy and program document such as acts of parliament, legislative instruments and annual reports.

### Sampling

Within each district, two communities each were selected for the community member FGD and key informant interviews. Criteria for selection were that in each district, one community should be within half an hour’s walking distance and the other an hour or more’s walking distance of the hospital (municipality) or health centre (rural district).

Within each of the study districts, a mix of purposive and snowball sampling was used to select respondents for key informant interviews to get the views of key stakeholders namely purchaser (NHIA district scheme staff), providers, district assembly (local government) and clients. NHIA district scheme staff interviewed, were the district scheme manager (head of the team), Public Relations Officer, Management Information Systems officer. Within each district assembly (local government), those interviewed were the district Chief Executive, Finance Officer, Coordinating Directors, Assembly men (elected community representatives of the district assembly), heads of unit and area committees and opinion leaders. Providers interviewed were the district director of health services, and health insurance claims officers at the health facilities. NHIA regional management staff were also interviewed.

In total 35 in-depth interviews were held. Interviews lasted between 30–70 min and were held in the offices and communities of the respondents. There were some rare situations where interviews were arranged and held at the homes of respondents for their convenience.

Community focus group discussions were held with adult groups of currently enrolled, previously enrolled but currently uninsured because of failure to renew, and never insured in each of the four communities. Participants were purposively selected to ensure a mix of male and female and range of ages between 18 and 82. Community FGD were also held with insured pregnant women. They were recruited from pregnant women attending antenatal clinics and from the study communities. Pregnant women who participated in the FGD were between the ages 17–45. All focus group discussions were held in the community in the dominant local language, Ewe, and lasted between 30 – 60 min. In all 12 focus group discussions of between 8 – 11 persons per group were held.

All Interviews were moderated by members of the research team assisted by research assistants who were recruited and trained for the purpose. Interviews were tape recorded in addition to the interviewers notes and transcribed verbatim into English so all team members could read the notes. All interviews and recordings were done with informed consent (Additional file 1).

### Data analysis

Qualitative data was analysed manually using thematic content analysis of the text around the study questions guided by the analytical framework for the study. Routine management information systems data on enrolment was analysed in Excel for percentages, patterns and trends.

## Results

### Who is enrolling in the NHIS?

Table 1 and 2 summarizes data from the two district schemes on new enrolment, renewals and total active membership as well as enrolment by category from 2010 – 2013. The data suggests that renewals of enrolment are increasing, while new enrolments are somewhat stagnant. The net result is a steady increase in total enrolment in both districts with a level of between 40 – 50 % at the time of the study. These observations from the data mirrored the perceptions of respondents in the primary data collection.

**Table 1:** Trends in new registrations and renewals by district from 2010 – 2013

Year	New registrations	Renewals	Active membership	Total pop	% Total pop. new registration	% Total pop renewal	% Total pop active membership
Municipal mutual health insurance scheme							
2010	40,824	42,736	83,560	271,881	0.15	0.16	0.31
2011	31,170	68,794	99,964	275,062	0.11	0.25	0.36
2012	28,268	90,401	118,669	278,280	0.10	0.32	0.43
2013	32,922	109,746	142,668	281,536	0.12	0.39	0.51
Rural district mutual health insurance scheme							
2010	6594	7426	14,020	64,404	0.10	0.12	0.22
2011	9036	10,901	19,937	65,158	0.14	0.17	0.31
2012	16,480	15,479	31,959	65,920	0.25	0.23	0.48
2013	10,082	20,811	30,893	66,691	0.15	0.31	0.46

**Table 2:** Registration by category in the two districts from 2010 – 2013

	2010				2011				2012				2013			
	New	Renewal	Total	% of total	New	Renewal	Total	% of total	New	Renewal	Total	% of total	New	Renewal	Total	% of total
Rural district mutual health insurance scheme																
Adult informal	1557	1748	3305	23.6 %	1655	2477	4132	20.7 %	2879	3267	6146	19.2 %	965	4046	5011	16.2 %
Aged	610	1049	1659	11.8 %	300	1401	1701	8.5 %	375	1565	1940	6.1 %	284	1624	1908	6.2 %
Children under 5	10	-	10	0.1 %	37	9	46	0.2 %	68	39	107	0.3 %	65	84	149	0.5 %
Dependent	2286	2563	4849	34.6 %	4115	3771	7886	39.6 %	5713	5601	11,314	35.4 %	4512	7695	12,207	39.5 %
Indigent	883	1301	2184	15.6 %	1573	2075	3648	18.3 %	5472	3421	8893	27.8 %	3114	5471	8585	27.8 %
Pregnant women	1091	309	1400	10.0 %	1210	579	1789	9.0 %	1280	892	2172	6.8 %	821	880	1701	5.5 %
SSNIT	144	418	562	4.0 %	133	572	705	3.5 %	82	707	789	2.5 %	51	739	790	2.6 %
Unknown category	13	38	51	0.4 %	13	17	30	0.2 %	611	5	616	1.9 %	270	272	542	1.8 %
Grand total	6594	7426	14,020	100.0 %	9036	10,901	19,937	100.0 %	16,480	15,497	31,977	100.0 %	10,082	20,811	30,893	100.0 %
Municipal district mutual health insurance scheme																
Adult informal	16,745	15,551	32,296	38.7 %	13,257	26,311	39,568	39.6 %	10,128	35,503	45,631	38.5 %	9125	41,486	50,611	35.5 %
Aged	3677	3946	7623	9.1 %	1080	6765	7845	7.8 %	578	7212	7790	6.6 %	538	7246	7784	5.5 %
Children under 5	40	5	45	0.1 %	207	30	237	0.2 %	289	194	483	0.4 %	367	425	792	0.6 %
Dependent	10,926	12,827	23,753	28.4 %	10,541	19,246	29,787	29.8 %	11,717	27,088	38,805	32.7 %	12,331	34,984	47,315	33.2 %
Indigent	3176	3175	6351	7.6 %	2457	5231	7688	7.7 %	2907	7397	10,304	8.7 %	8739	11,025	19,764	13.9 %
Pregnant women	2258	1221	3479	4.2 %	2421	2288	4709	4.7 %	2104	3104	5208	4.4 %	1435	2918	4353	3.1 %
SSNIT	3305	5879	9184	11.0 %	1176	8877	10,053	10.1 %	543	9794	10,337	8.7 %	373	10,560	10,933	7.7 %
Unknown category	697	132	829	1.0 %	31	46	77	0.1 %	2	109	111	0.1 %	14	1102	1116	0.8 %
Grand Total	40,824	42,736	83,560	100.0 %	31,170	68,794	99,964	100.0 %	28,268	90,401	118,669	100.0 %	32,922	109,746	142,668	100.0 %

The Ghana NHIS law (Act 852 and the preceding Act 650) all define out of pocket premium exempt categories. These include children under 18, the elderly defined as those over 70, persons with mental disorders, pregnant women and the indigent (too poor to pay); and also SSNIT contributors and SSNIT pensioners because of the direct deduction of 2.5 % of social security contributions. (Act 852 section 29). Almost all the categories are “demand” driven in the sense that the clients are the ones who define themselves based on their demographics (age) or situation (e.g., pregnancy) that are easily verifiable. The category of indigents, however, is supply driven in the sense that apart from LEAP beneficiaries; it is the district scheme management that ultimately takes the decision as to who is an indigent.

### Why are people enrolling (or not)?

**Fig. 1:**
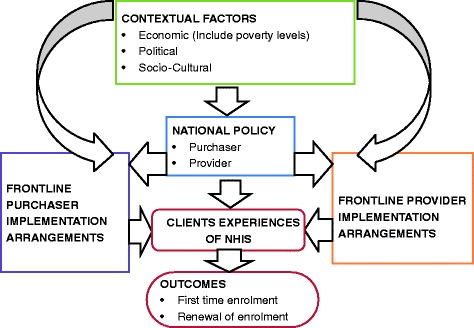
Summary of factors affecting enrollment

Reasons why people are enrolling or not in the NHIS that emerged from our data could be classified as factors related to purchaser, provider, client and context as we theorized at the beginning. However, from a more analytic perspective of organizing our data to help provide answers to the “why” of observed enrolment patterns we found it more useful to reorganize the purchaser and provider factors into those predominantly influenced by national policy and program arrangements and those more influenced by peripheral implementation arrangements of frontline (mainly district level) purchaser and provider staff. Moreover, our observations that could be classified as “client factors” such as unexpected and unaffordable out of pocket payments; and opportunity costs of enrolling in and using the NHIS (mainly time and transport costs) that deterred enrolment were more accurately client experiences triggered by one or more of the factors classified as national policy or frontline provider and purchaser implementation arrangements. The factors  worked in an inter-related manner rather than in isolation. We therefore organized our findings into an explanatory framework as summarized in Fig. 1.

### National policy

By national policy, we refer to the national level NHIS program decisions, legislative and administrative arrangements, rules and procedures that dictated how the scheme should be implemented. These arrangements in stipulating what should be done and how, created intended as well as unintended incentives and sometimes conflicts in context, for client, purchaser and provider behaviour that affected enrolment positively or negatively. They included factors such as how the scheme is financed, who pays and who benefits, enrolment rules, how providers are paid and the prescribed benefit package.

### Perceptions of who pays and who benefits

Some of the targeted out of pocket premium exemptions contributed to a perception among insurance staff and community leaders that the scheme was predominantly for the benefit of pregnant women and children under 18 and not so much for other sections of the population. It was also seen to be more attractive for these categories to preferentially enrol.

*“(…) under 18 and pregnant women are enrolling because of the policy put in place.” (Scheme Staff, Municipality)*

Though the “indigent” are also out of pocket premium exempt there was no perception that they were benefitting more from the scheme than others. Their numbers enrolled in the scheme were very small in both districts and only jumped up dramatically in 2013. Insurance scheme staff felt this was because the definition of indigents lacked clarity in the national policy.

*“Indigents are also not enrolling because the indigent clause is so stiff. The pointers to who an indigent is were not clear.” (Scheme staff)*

### Annual enrolment and renewal

Community leaders, local government members and insurance scheme staff observed that most users do not remember the expiry dates of their insurance cards. Often it was only when they ended up in hospital for a health problem that they became aware their cards had expired.

*“I think most of the people when their card expire, they don’t even know, they get to the facility to access medical attention before realizing that their cards have expired.” (local government staff)*

### SSNIT contributor and pensioner disinterest

District scheme staff observed that for several different reasons SSNIT contributors and pensioners were not enrolling much in the NHIS. SSNIT contributors generally have relatively more stable jobs and therefore predictable income. Some of those who could afford it preferred to go to private facilities rather than the Government facilities covered by the NHIS card, because of perceptions of better quality of care in private facilities. Having to go to the scheme offices and join the queues to pay a registration fee to renew their cards despite the deduction of 2.5 % of their social security contributions into the National Health Insurance fund was also a disincentive to enrolling.

### Non SSNIT subscribers out of pocket premiums

Another design challenge had to do with the premiums and renewal fees for non SSNIT subscribers. In theory these rates were supposed to be on a sliding scale proportional to wealth and ability to pay. In practice there was no robust sliding scale design available to assess ability to pay and premium rates were effectively flat for all subscribers irrespective of their income levels. In the rural district many community members, as well as scheme and local government staff, felt that the premiums and registration fees were higher than the ability of community members to pay; and therefore a barrier to enrolment. This was less of a challenge in the urban municipality.

*“…….they don’t have money because most of them are peasant farmers and only farm to eat*.” *(Local government staff, Rural District)*

The NHIS allows individual rather than mandated community or family registration. Some respondents were of the opinion that the non SSNIT subscribers, especially those between the ages of 18 and mid 40s were not enrolling because they felt their risk was low.

*“The informal sectors are not enrolling because they happen to be the strong group in the age brackets (above 18 to mid-40s) where they hardly fall sick, so they don’t see the reason why they should come for an insurance card.” (Former Scheme Manager)*

### Opportunity costs

The opportunity costs, mainly time and transport, of having to actively go to a center to enroll as well as the direct costs (out of pocket premiums) to be paid at the time of enrolment also contributed to the disinterest of the healthy whether SSNIT or non SSNIT subscriber to enroll in the absence of compulsion.

### Claims reimbursement

A major problem mentioned by purchaser and provider staff were the delays in provider claims reimbursement and its effects on provider responsiveness to clients, client satisfaction and ultimately enrolment. The National Health Insurance Fund (NHIF) is a centrally pooled and managed fund with funds flowing from the Ghana Revenue Authority which collects taxes and the SSNIT through the Ministry of Finance to the NHIA to pay providers. Releases to frontline providers are the end of a chain that starts at national level. The timing of the releases was largely beyond the control of frontline purchaser staff in the district schemes. The long delays affected implementation by their effects on provider coping behaviour. This is discussed further under frontline provider implementation factors.

### Identity card processing and issuing

Respondents in both districts identified delays in processing and issuing NHIS identity cards as another reason for people losing interest in enrolment. The rules as to what kind of identification is used to guarantee benefits are dictated by national level policy arrangements and guidelines.

At the time of the study the NHIA had introduced an NHIS instant biometric card system in other regions to resolve the delays. Implementation had not yet started in the Volta region. Some scheme staffs felt the planned biometric registration system would be key to ameliorating this problem. However others were worried because of widespread negative reports of the system delaying rather than speeding up card issue from colleagues in regions where biometric registration had started.

### District NHIS office staff and logistics

The district scheme offices were faced with challenges related to inadequate funding, staff and logistics, that affected their performance. Allocation of funding, staffing and logistics in the district scheme offices is largely controlled centrally.

*“(…) look at this office, it is only now that we are moving to a new place just because we are not able to raise enough fund to do things for ourselves (…) we don’t have enough money to run the office.” (Health Insurance staff, Rural district)*

### Health facility availability and resources

Resources for health facility construction and decisions on locations of health facilities in the public sector are often centrally controlled with resources availability for infrastructure development inadequate in relation to needs. The absence of a district hospital with basic services such as laboratory tests, scans etc. was a major barrier to registration and renewal in the rural district. In both districts, health facilities available were inadequate for the numbers seeking care. There was sometimes congestion in waiting areas, long waiting times and inadequate and overburdened health staff.

*‘’Where I visit the space is small and the equipment are also not adequate. You also spend a lot of time waiting because it is the same doctor attending to everyone (…)” (Community member Urban district)*

People also had unmet expectations about the content of service related to the skills and capabilities of staff. In many of the FGDs; community members perceived quality of care as including seeing a *‘certified medical doctor (…)’* and/or *‘being given lots of medicines and an injection for the treatment of an ailment (…)’*. Health workers and the scheme officers were aware of these perceptions and expectations.

*‘(’…). when there is no doctor in the health facility they (community members) find it very difficult to have that confidence to go to seek health at the facilities; so for now I think there should be a district hospital that is run by a medical doctor and not a medical assistant or physician assistant because some of the diagnosis it means we have to refer.” (*Scheme Manager Rural district)

In the better served urban area FGD, the frequently expressed problems with staff skills and referrals in the rural area FGD came up less as issues of concern.

### Frontline purchaser implementation arrangements

Frontline purchaser implementation arrangements for the purposes of this study are defined as the actions and inactions of district staff of the national health insurance authority responsible for implementation of the scheme. Within the context of the policies, rules and regulations stipulated for the NHIS, they had significant power to inhibit or facilitate the achievement of the objectives of the national health insurance scheme in implementation by using discretion. Issues related to decision making and executing at the level of frontline purchaser staff included deciding who was an indigent, formal and informal payments for ID cards, and responsiveness to clients (customer relationships).

### Formal out of pocket payments in scheme offices

Some scheme staff observed that local processing fees introduced for SSNIT pensioners because their former employers were not paying these fees seemed to be acting as a barrier to enrolment.

*“The pensioners: Initially they were not charged anything but we realized that those institutions who promised to pay their processing fee were not paying, so we decided to charge them processing fee of 2.00. Some of them have refused to pay the 2.00 so they don’t come to register.” (Scheme Staff, Rural district)*

### Informal out of pockets payments in scheme offices

Community members in both districts mentioned informal payments during enrolment as one of the hindrances to enrolment. NHIS registration agents as well as some insurance staff were alleged to be charging under-the-table fees for services, which were supposed to be free.

*"(…) they say your card is not working again so you have to renew it. They will say bring something before they renew it, and because you too you don't have money you go; and you are sick but because you don’t have money you can't renew it." (Insured member, Rural Community)*

### District scheme office responsiveness to clients

Another purchaser implementation challenge mentioned in both districts was poor frontline purchaser staff customer relations. Clients in the FGD shared negative experiences in the scheme offices such as: *"The officers shout at you when you are young.", "Even when you are old they shout at you.", "They come and insult us in English."* An opinion leader suggested that: *“The staff of the insurance scheme must be schooled on customer relations.” (Rural & Urban Community FGD)*

### Interpretation of rules

Some scheme staff were praised by community members for using their discretion to ensure flexibility in interpretation and enforcement of national level policies and regulations in ways that enhanced enrolment. Conversely others were criticised for their lack of flexibility in enforcing rules to the detriment of enrolment. Scheme managers sometimes found themselves in dilemmas where to use discretion and bend the rules to encourage enrolment might be at the risk of losing their jobs, for example, ﻿and yet in the context of low levels of literacy to strictly enforce rules such as a three month waiting period for clients who forgot to renew their card on time sometimes came across as inhumane and creating undue hassle for beneficiaries.

*“I never applied the default policy (…) and I was ready to answer any query to that effect (…) because of illiteracy on the part of the client. There are policies you can implement in certain places conveniently, but in certain areas you must give it a human face.” (Scheme Staff)*

A rule whose vagueness left a lot of room for discretion in interpretation was that guiding the decision on “who is an indigent?”.

*“The pointers to who an indigent is were not clear. It was quite recently that social welfare department gave some guidelines on how to identify an indigent that we have started registering them.” (Scheme Staff)*

Though indigents are exempt from premium payment the clarity, specificity and sensitivity of operational criteria for their identification is a challenge. Act 650 (section 38) required that *“The Minister on the advice of the Council shall prescribe a means test for determining persons who are indigent.”* and *“A district mutual health insurance scheme shall on the basis of the means test, identify and keep a list of members registered with it who are indigent.”* Act 852 (section 29) states “*The categories of persons exempted from the payment of contributions under the scheme include (…). A person classified by the Minister responsible for Social Welfare as an indigent.”*

Discretion was used to keep people out as well as to swell up numbers.

*“…I will keep my indigent numbers as a banker…if I see that at the end of the month I am not getting results, I will go to one village and then grab all their cards and see if they are indigents and then get the numbers up.” (Scheme Staff)*

### Frontline provider implementation arrangements

#### Health service provider responsiveness to clients

The responsiveness of the services to the social aspirations of people such as treatment with respect and dignity, confidentiality, autonomy in decisions concerning their own health and non-discrimination also affect their acceptability. Perceptions of language barriers and religious discrimination were among responsiveness related reasons some respondents gave as a disincentive to seek health care that affected NHIS enrolment even when the services were available.

*‘’Health insurance is not serving the purpose for which it was done. When we go to the hospital and they see the “mayafi” (Hijab) they end up insulting us that as for Muslims they like to give birth so even when they don’t charge others they will charge us (…)” Urban (Community member)*

*‘’Sometimes too they assume everyone speaks Ewe and English so when you can’t speak any they get angry at you.” (Community member)*

Closely related to the issue of responsiveness as a barrier was what was described as “Attitude of health workers”.

*‘’When you come to the hospital and you are in labour some staff do not attend to us, some too come to work too late, they come when patients are already waiting (…)” (Pregnant woman)*

### Formal out of pocket payments in provider facilities

Patients having to pay out of pocket for several services including medicines even when insured was common in some facilities and sometimes served as a major source of client discontent and disinterest to enroll in the NHIS.

*“Here lies the case: we talking about health insurance here but when you go to the hospital (…) they don’t give you the actual medicine, they write it for you to go and buy from the drug store. Mine (insurance card) is four years (expired) now. I don’t want to renew it. I will rather take money to go to the drug store to buy the medicine than to renew it.” (Uninsured Client, Urban community)*

Community members also reported being asked to make top up payments with the explanation that NHIS reimbursement rates were inadequate. Long delays of several months in provider reimbursement were blamed by providers as well as insurance scheme staff for reinforcing what providers labelled as “coping” or “managing” behaviours such as charging clients co-payments, top up payments or balance billing; and disinterest in or inability to stock adequate quantities of medicines covered by the NHIS.

*“(…) you see sometimes the facilities blame us for not paying claims on time, for owing them so much that is why they are not able to stock drugs (…)” (Scheme staff, Rural District)*

### Informal (under the table) charges

Outright under the table charges and extortion were also reported in the community interviews. The qualitative nature of the study did not enable quantification of the extent. Though not directly related to the NHIS or unique to NHIS clients, they were a disincentive to use health facilities and therefore to enrolment.

*‘’Some are given drips (Intravenous infusions) when they are due for delivery, others too they are not given drips. But what some of the nurses do is that even when you are not given drips they will still ask you to go to the dispensary and collect it. When you bring it to them they keep it and sell to another. They also will take 2 geisha soaps and 8 cedis from you after delivery.” (Pregnant woman community member)*

### Contextual factors

Poverty levels and dependency ratios were both high, and this affected the ability to pay premiums especially in the rural district.

*“Some of them too have a large family size. So if a peasant farmer whose income is seasonal – and mostly when they come for the card they come as a group – so if the card is expired within this period it is difficult for them for renew because of the family size.” (Scheme Management staff)*

District Scheme management staff observed that bountiful harvests sometimes translated into increased enrolment. However in the pre-harvest period, the rains and related intense farming activity sometimes meant limited time for people to spend at scheme offices to enrol. In some cases people also moved to their villages to farm, making it even more difficult for them to come to the scheme offices.

In the rural areas the poor road networks, further complicated access to scheme offices and health facilities by increasing the travel and time costs and thus serving as a disincentive to enrolment related to economic context.

Politicization of the NHIS was mentioned by many respondents as a factor that affected enrolment negatively or positively depending on the details. A district scheme staff gave an example of how they sometimes tried to use the political interest in the scheme positively to enrol clients.

*“…what I did was to get the MP to vote some money purposively to target the informal sector. I decided the MPs vote will not pay the premium in full so what we did was to ask them to contribute about 42 % of the amount; and people patronized. We had long queues (…) we made great impact.” (Scheme Management staff)*

On the negative side, the political debates on which political party owned the national health insurance scheme were perceived to have affected enrolment in the region especially at the initial stages. At the time of the introduction of the NHIS in 2003, there were many strong activists of the then opposition party, in the region, who did not want to have anything to do with the scheme. On the positive side, when this party with a strong following in the region came to power in 2009, the sense of ownership increased and with it, interest in enrolment.

Respondents however mentioned that over time they saw a growing sense that the NHIS is non partisan and belongs to residents of Ghana rather than any political party.

*“….people try to play some sort of politics with it but I want to say that where we have gotten to now, so far national health insurance is concerned, it is non-partisan and anybody that will make it fail, Ghanaians will never forgive the person. Even posterity will never forgive the person.” (Scheme management staff)*

The main socio-cultural factor mentioned as affecting enrolment was a small religious sect called “The First Church”, whose members refuse to enrol because of their beliefs against the use of biomedical care when ill. Its impact did not appear to be significant.

### Effects on clients experiences of NHIS

The clusters of National policy, Contextual, frontline purchaser and provider implementation arrangements interacted with each other to produce client experiences of the NHIS. Some of these were positive, with some clients reporting satisfaction with having used the NHIS card, receiving prompt attention and not having to pay any bills. Others were negative effects and ended up becoming disincentives to enrolment in the NHIS.

For example, the time and travel costs of going to the scheme offices and outlets to enrol or renew, that were sometimes as major as a whole day or even more could make enrolment very expensive. If, additionally, a client experienced co-pays, balance billing and under the table charges in a health facility, it added up to even more days of wages spent in accessing care under a “free NHIS”.

These factors also sometimes combined to create a negative effect of experienced discrepancies between what was officially stated as covered by the NHIS policy and what clients experience as covered by the NHIS in actual use. It was commonly stated by decision makers that the NHIS operates a generous benefit package covering about 90–95 % of common diseases in Ghana. The impression clients got from these statements was that once insured, all or at least almost all services would be free at point of service use. When on presenting at health facilities with their cards they met various co-pays, non-available medicines that had to be purchased outside etc. as already described; the result was often disenchantment with, and mistrust of the NHIS and its promises.

*“The reason I enrolled is that we were promised that when you are able to enrol you have access to everything when you attend the hospital. But there have been times when you go to the hospital you are told that certain conditions are not covered by the card or we don’t have this or that drug (…)” (Insured community member, rural district)*

## Discussion and conclusions

This study aimed to understand why enrolment in the Ghana NHIS has stagnated and the implications for policy and program design and implementation towards universal population coverage. We explored who is enrolling (or not) and barriers and enablers that explain the observations. Rather than any single factor, we found a multiplicity of interacting factors. These factors can be broadly classified as related to national policy and program arrangements, implementation arrangements and context, and summarized in an explanatory framework as in Fig. 1.

Attaining universal population coverage with health insurance took several decades in lead countries such as Germany and Japan. Germany, where classical social health insurance originated, achieved UHC about twelve decades after the first sickness funds became operational [20]. Japan, who used the German model as a blue print achieved UHC about four decades after legislation was passed. Times for recent achievers such as South Korea, Thailand and Taiwan have been much shorter. South Korea achieved UHC in 1989, a little over a decade after its revised health insurance law started implementation in 1977 [21]. Thailand achieved UHC in 2002, a little over two decades after its medical welfare schemes were initiated [22, 23].

In Africa, though a UHC trail blazer with the passage of its national health insurance law in 2003, Ghana continues to face several challenges. There are lessons from the experiences of South Korea and Thailand to some of the challenges to population coverage this study has uncovered. Both countries achieved UHC while they were still classified as LMIC. South Korea’s Gross Domestic Product per capita in 1977 when they passed their revised health insurance law was estimated at US$ 1042; and US$ 5430 when they finally attained UHC in 1989 [22]. Thailand had a Gross National Income per capita of US$ 390 when their medical welfare scheme started in 1975 and US$ 1900 in 2002 when they finally attained UHC.

Experience from CBHI schemes [15, 24] as from Ghana’s NHIS suggest that when enrolment and renewal in health insurance, is predominantly voluntary, attaining universal coverage is challenging. Ways have to be found to introduce compulsion. Like many other LMIC, Ghana’s large non formal sectors and the non-existence of a universal citizen registration database, is part of the challenge of effecting compulsory enrolment. Ghana’s choice of a single payer mixed tax and contributory insurance scheme with some out of pocket premium payment was based on historical and pragmatic as well as technical considerations [25]. On the other hand, its decision to implement individual registration is a weakness that could be changed.

South Korea, from the beginning of its efforts to attain UHC adopted family based registration. Formal sector employee registration in the workplace was done with their dependents as members of the scheme of the household head. Regional arrangements for registration of the non-formal sector also mandated household registration.

Public and private formal sector employees in Ghana form about 5 – 10 % of the population and are relatively easily found on the SSNIT and other databases. Systems to allow employer employee contributions deducted at source for the contributor as well as dependents can be set up. Given the average household size of 4 in Ghana, registering dependents with employees will ensure coverage of another 30 – 40 % of Ghana. It will also reduce for SSNIT contributors, pensioners and their dependents the current time and travel costs involved in NHIS enrolment. There should be room to negotiate an increased percentage deduction to allow dependent coverage. In Ghana employer employee contributions from SSNIT subscribers are 2.5 % of contributions to the social security fund. This is low by international standards. For example, contribution rates in South Korea have been about 5 % of wages on average [22]. Rates in Taiwan are similar [26].

Identification of beneficiaries is critical. For efficiency reasons, it is worth exploring how to strengthen and use already existing identification systems or merge systems rather than create parallel systems. Ghana’s creation of a special NHIS card, as this study shows, has become to some extent a bottleneck. Thailand used their already existing national identification system for clients to gain access to the Thailand NHIS. Learning from this model can remove the enrolment barriers posed by the time and travel costs of getting a special NHIS card.

Removing the work of special card issuance and enrolment of formal sector subscribers and their dependents from the district scheme offices will also allow more resources of the district schemes to be focused on identifying and reaching the poorest of the poor. Strategies such as door to door enrolment processes make it easier to get an idea of living standards and incomes for targeting purposes. South Korea used a community based system of means testing to help it identify what contributions levels would be reasonable for non-formal sector enrollees. Currently identifying the poorest of the poor is a major challenge to implementing the indigent exemptions policy under the NHIS.

Ghana’s macro economy, like that of many other LMIC is an important contextual factor influencing health system resource availability. Thailand, South Korea and Taiwan who have attained UHC in recent times experienced economic growth over the period of their transition. This made resources available to support UHC once it was a priority government agenda. Advocacy for UHC must be accompanied by awareness raising about the importance of economic growth; and policies that stimulate economic growth in LMIC. Though harder to deal with than adjusting implementation arrangements, it is important to keep attention on the link between national wealth and the ability to implement a public policy focus and commitment to UHC.

This study did not set out to investigate street level bureaucracy theory. However, some of its findings are explained by this theory. Lipsky [27] in his exploration of how and why some types of public service agencies perform contrary to their own rules, coined the term street level bureaucrats to describe “*Public service workers who interact directly with citizens in the course of their jobs and who have substantial discretion in the execution of their work*”. He found that the combination of considerable discretion in decision making, regular contact with citizens and relative autonomy from organizational authority, meant that the sum of the individual decisions, actions and inactions of street level bureaucrats becomes agency policy in practice. At the same time, their conditions of work tend to induce coping behaviors that modify policy in implementation and produce results different from stated agency objectives. These conditions of work include inadequate resources, and demand for services that tend to increase to meet supply. Agency goals may be ambiguous, vague or conflicting. Clients are non-voluntary with limited alternatives and control over frontline worker behavior. It is vital that policy and program formulation for UHC adapts principles in context through careful observation, analysis and responsiveness to actual and potential frontline purchaser and provider incentives and related behaviour that may be generated at implementation levels. Otherwise avoiding policy failure and implementation gaps can be very difficult.

## Conclusions

Ghana’s continuing challenges with attaining universal population coverage in its NHIS are related to policy and program design features as well as implementation factors. Within any given level of resource availability, to attain universal population coverage with a contextually affordable package of essential care requires policy and program design that ensures that enrolment in national health insurance is effectively compulsory rather than voluntary in implementation. It also requires careful attention and responsiveness to actual and potential subscriber, purchaser and provider (stakeholder) incentives and related behaviour generated at implementation levels. There is much that Ghana and other countries in sub-Saharan Africa can learn, not only from the current study of Ghana’s experience to date; but also from countries such as South Korea and Thailand in the Asian region who have attained near Universal Population coverage and did so while they were still in the category of lower middle income countries.

## Additional file

Additional file 1: Data collection tools-The Universal in UHC. Primary data collection tools. Informed consent form. Focus Group Discussion and Key Informant interview guides. (DOCX 136 kb)

## Abbreviations

LEAP: Livelihood empowerment against poverty
LMIC: Lower and middle income country
NHIS: National Health Insurance Scheme
NHIA: National Health Insurance Authority
SSNIT: Social Security and National Insurance Trust
UHC: Universal Health Coverage
