# Supplementary material for: The “Universal” in UHC and Ghana’s National Health Insurance Scheme: policy and implementation challenges and dilemmas of a lower middle income country
Source: BMC Health Serv Res. 2016 Sep 21;16:504. doi: 10.1186/s12913-016-1758-y (PMC5031274; doi:10.1186/s12913-016-1758-y)
Supplement: Additional file 1: — Data collection tools-The Universal in UHC. Primary data collection tools. Informed consent form. Focus Group Discussion and Key Informant interview guides. (DOCX 136 kb) [file 12913_2016_1758_MOESM1_ESM.docx]

Primary Data Collection Tools

The Universal in UHC and Ghana’s National Health Insurance Scheme: Policy and Implementation Challenges and Dilemmas of a lower middle income country

# Informed consent form

## Purpose of the research

The Research and development Directorate of the National Health Insurance Scheme (NHIS) and the Korean Foundation of International Healthcare (KOFIH) are conducting a survey on health insurance in the Volta Region. We are here today to ask you about health insurance issues in your community. A number of districts in Ghana, including your district, have low enrolment in the health insurance scheme. To improve enrolment we have come to talk about what you and other community members do with regards to health insurance enrolment. We are conducting several meetings with people like yourself to find out your experiences with the scheme and your views about how we can effectively tackle the problems of health insurance services in our communities.

The information you give us will assist us make plans for improvement. Your experience and opinions are very important in this endeavour.

## Procedures

If you are willing to be part of this study, we will ask you a range of questions. You are required as a member of the community to give us your opinion on the questions that we will pose based on your personal experiences and your experience within the community. You are free to speak your mind about how you think and feel about any issue that is raised here. There are no wrong or right answers. You are free to discontinue with the discussion or refuse to answer any issue you feel uncomfortable to talk about and this will not affect you in anyway. **All of your comments will be recorded only for the research, not for collecting personal information.**

## Risks and discomforts

There may be a minimal risk that some questions will be personal. We do not expect this to happen. However, should that happen and you do not feel comfortable to share any personal or confidential information with us, you may decline to respond to those questions. The survey will take about one hour.

## Benefits

Participating in this study may not benefit you directly, but it is likely to help us find out how we can engage population enrolment for National Health Insurance Scheme at the community level.

## Confidentiality

Whatever you say will be confidential so feel at ease to express your opinion. Your name will not be recorded on any form or be used in analysis or dissemination of results. The information you give us will be put together with what others tell us so it cannot be linked to you. The information that is obtained from the surveys will be used only in a form that cannot be identified with you.

## Incentives

Your participation in this study is voluntary and you will not be paid for this discussion.

Thank you.

## Who to contact:

For further information or clarifications you may contact **Prof. Irene Agyepong on telephone number 0244862665 or on email iagyepong@hotmail.com.** Also you can contact ethics committees review administrator (Hannah Frimpong 0243235225) if you need.

## Interviewer introduction text:

Hello. My name is __________________________, and I am working with the ***Ghana National Health Insurance Scheme***. We are conducting surveys on the health insurance scheme in Ghana, and your participation in the community would be most appreciated. I will ask you several questions about this service in your community. Your response will help us to plan health insurance scheme on improving community people’s health. The survey will take 40 – 60 minutes to complete. **All of your comments will be recorded only for the research, not for collecting personal information.** Any **personal** information you give will be kept strictly confidential and will be used for a final report and additional program development in the future.

Participation in this survey is voluntary and you can choose not to answer any particular question or all of the questions. However, we hope that you will participate in these interviews because your views are important. Do you have anything to ask before we start?

*(Please take a few minutes to answer any questions from the respondent)*

**Interviewer:** Do you agree to participate in these interviews?

| Respondent **AGREES** to be interviewed → | **Signature or Thumbprint of respondent:** | **_______________________________** |
| --- | --- | --- |
|  | **Date:** | (DD/MM/2014) |
| Respondent **DOES NOT AGREE** to be interviewed → | **Reschedule interviews?** | ❑YES ❑ NO |
|  | If **YES** → **Date:** | (DD/MM/2014) |
|  | **Time:** | **__________________** |
|  |  |  |
|  | **Signature of interviewer:** | _______________________________ |
|  | **Date:** | (DD/MM/2014) |
|  | **Time started:** | **__________________** |

# FGD & In-depth interview guide for community members

Never insured and previously insured and also community leaders (Chief, Assembly man/woman, Unit/Area committee member

1. What do you think are the health service needs of the people in your community and district?
2. How do people meet those needs?
3. Why?
4. Are you satisfied with the options for meeting your needs? Why or why not?
5. Whose needs are adequately met and whose are not? Why?
6. If you have ever insured before, why did you insure?
7. Why did you not renew your insurance?
8. If you have never insured why?

# FGD guide for insured and uninsured pregnant women

Never insured and previously insured

1. What do you think are the health care service needs of pregnant women in your community? For example infrastructure, service delivery, quality of care, medicines, HR etc. *(Probe for individual health care needs)*
2. How do you meet those needs?
3. Why these options? (*Probe for unmet needs under NHIS)*
4. Are you satisfied with the options for meeting your needs? Why or why not?
5. Have you ever been insured?
6. If YES, why did you insure and if NO why not? (*If yes, probe for perceived enrolment benefits and enrolment barriers on the other hand*

What do you think can be done to ensure people enrol or continue to be on the scheme? *(Probe for NHIS specific duties)*

# FGD guide for insured and uninsured community members

Never insured and previously insured and also community leaders (Chief, Assembly man/woman, Unit/Area committee member

1. What are your individual healthcare needs?
2. What do you think are the healthcare service needs of members of this community? For examples infrastructure, service delivery, quality of care, medicines, HR etc
3. How do you meet those needs?
4. Why these options?
5. Are you satisfied with the options for meeting your needs? Why or why not?

*Insured*

1. Have you ever been insured?
2. Why did you insure?
3. Would you recommend NHIS to another person? If yes, why and if no, why not?
   1. What do you think can be done to ensure your continued enrolment unto the scheme? *(Probe for NHIS specific duties)*

*Uninsured*

1. Have you ever been insured?
2. If yes why have you not renewed your insurance?
3. If NO, why have you not been insured?
   1. What do you think can be done to ensure you enrol unto the scheme? *(Probe for NHIS specific duties)*

# In-depth interview guide for community leaders

Community leaders (Chief, Assembly man/woman, Unit/Area committee member, Focal persons)

1. What do you think are the healthcare service needs of the people in your community? For examples infrastructure, service delivery, quality of care, medicines, HR etc
2. How do they meet those needs?
3. Why do they take these options?
4. Are they satisfied with the options for meeting their needs? Why or why not?
5. Whose needs are adequately met and whose are not? Why? *(Probe for these categories; pregnant women, women, men, adolescents, children, aged, physically challenged, mental patients, NCDs, CLLC etc)*
6. In your opinion what healthcare needs are adequately met by the NHIS and what are not? (*Probe for prospect for enrolment if NHIS met these needs)*
7. Per your observation, do you think people enrol onto the NHIS scheme in your community? If Yes why and if No why not?
8. Per your observation, how would you describe services rendered to insured and non-insured people at the facilities? (*Probe for differences)*
9. What do you think can be done to ensure they enrol or continue on the scheme?

*(Probe for NHIS specific duties)*

# In-depth interview guide for Scheme management staff

Scheme manager, Public Relations Officer, Management Information System officer

1. To your observation, what have been the trends in NHIS enrolment in this district over time? Do you have data to support your answer? If so could we have a look at it
2. To your observation, who is enrolling in the NHIS In this district, who is not and why? Again do you have data to support your answer?
3. Why do you think people do not renew their enrolment in the NHIS in this district?
4. How big a problem is it?
5. What are the health service needs of the general population in relation to the NHIS HC
6. Whose needs are met and whose are not
7. Why are their needs met and why not in relation to NHIS
8. If you were asked to suggest how to get everybody in this district insured and keep them insured what would be your suggestion?

# In-depth interview guide for district assembly members (include DDHS)

DCE, DCD, DDHS, Presiding member, frontline worker @ a facility (Health worker, NHI worker at the facility

1. To your observation, what have been the trends in NHIS enrolment in this district over time? Do you have data to support your answer? If so could we have a look at it
2. To your observation, who is enrolling in the NHIS In this district, who is not and why? Again do you have data to support your answer?
3. Why do you think people do not renew their enrolment in the NHIS in this district? (*Probe for relationship between unmet healthcare needs and non-renewal of enrolment)*
4. How big a problem is it?
5. What are the health service needs of the general population in relation to the NHIS HC
6. Whose needs are met and whose are not
7. Why are their needs met and why not in relation to NHIS
8. If you were asked to suggest how to get everybody in this district insured and keep them insured what would be your suggestion?
9. Per your observation, how would you describe services rendered to insured and non-insured people at the facilities? (*Probe for differences)*
